# Lack of Evidence for Blood Pressure Effects of Caffeine Added to Ibuprofen

**DOI:** 10.2174/1574886317666220414125027

**Published:** 2023-01-01

**Authors:** Anette Lampert, Robert Lange, Thomas Weiser

**Affiliations:** 1 CHC Medical Affairs, Sanofi-Aventis Deutschland GmbH, Frankfurt am Main, Germany

**Keywords:** Caffeine, ibuprofen, blood pressure, heart rate, adverse effects, drug metabolism

## Abstract

**Background:**

Caffeine enhances the efficacy of non-opioid analgesics. Data on the cardiovascular health effects of caffeine intake are controversial, and studies on the cardiovascular effects of medical caffeine use are lacking.

**Objective:**

The study aims to explore the cardiovascular effects of an ibuprofen/caffeine combination in comparison to ibuprofen alone.

**Methods:**

Secondary analysis of a previously reported bioequivalence study of a single dose of a fixed dose ibuprofen/caffeine combination (400/100 mg) *vs*. ibuprofen alone in a randomized, cross-over design in 36 healthy volunteers. Plasma catecholamines were analyzed to enhance mechanistic interpretation of the data.

**Results:**

After exclusion of 10 protocol violators (pre-dosing intake of caffeine), vital signs were comparable over a 24-h period in the absence and presence of caffeine. Plasma catecholamine levels were also comparable.

**Conclusion:**

These data do not support the hypothesis that occasional intake of a small dose of caffeine as part of pain medication imposes a health risk due to vital sign changes. Based on the proven increase in efficacy, the addition of caffeine to non-opioid analgesics such as IBU has a favorable risk/benefit profile for occasional use.

## 1 INTRODUCTION

Caffeine (CAF) enhances the efficacy of non-opioid analgesics in the management of pain in general [1] and that of ibuprofen (IBU) in particular [2]. This synergism has led to the development of fixed dose combinations of IBU/CAF. On the other hand, reviews and meta-analyses have shown that CAF consumption can increase blood pressure (BP) and be associated with cardiovascular events [3, 4]. The overall data suggest that CAF effects on BP are more easily detected in clinical acute studies than in large cohorts and mostly occur with high doses of CAF. However, the overall data are complex and difficult to interpret due to confounding factors such as gender, age, lifestyle factors, and genetic differences in CAF metabolism [4]. These data necessitate balancing the beneficial effects of CAF with the efficacy of analgesics with possible adverse effects on BP and cardiovascular health.

The existing data on CAF intake and BP regulation are largely based on chronic CAF consumption as it occurs in habitual drinkers of caffeinated beverages, including energy drinks; in contrast, therapeutic use of IBU/CAF combinations occurs largely sporadically and/or for a short time, *e.g*., in the treatment of headache [1, 5]. Therefore, whether data based on recreational ingestion of CAF can be extrapolated to its medical use in combination with non-opioid analgesics remains unclear.

Considering this background, we have analyzed data from a recently reported study in healthy volunteers [6] to explore the short-term BP effects of an IBU/CAF combination compared to IBU as monotherapy. For a better mechanistic understanding, we related the observed effects on vital signs to CAF plasma concentrations and adrenaline and noradrenaline.

## 2 MATERIALS AND METHODS

Our analyses are based on data obtained as part of a previously reported study [6] registered on clinicaltrials.gov as NCT02629354. The study protocol was in line with the Declaration of Helsinki and Good Clinical Practice guidelines and Directive 2010/63/EU and approved by an independent ethical committee (Independent Ethics Committee, Faculty of Health Research Division, Bloemfontein, South Africa, approval date 4 November 2015). All participants provided informed written consent prior to the start of the study. Briefly, 36 subjects were enrolled and randomized in a 2-period cross-over study and randomly assigned to one of two treatment sequences with a washout period of at least 6 calendar days: they received either 400 mg IBU (administered as 684 mg IBU lysinate) or 400 mg IBU (administered as free acid) in a fixed-dose combination with 100 mg CAF (IBU/CAF) as a single oral dose following a defined breakfast. However, 10 subjects were excluded due to protocol violations (see below). Thus, the pharmacokinetic population included 25 male and 11 female healthy subjects (pharmacodynamic population; mean age ± SD 28.0 ± 7.3 years; body weight 70.4 ± 10.2 kg; body mass index 24.1 ± 3.1 kg/m^2^). The pharmacodynamic population was defined as all subjects within the pharmacokinetic population who had at baseline and at least one postdose measurement available for plasma catecholamines and included 21 subjects (subjects 1, 17, 18, 19, 20, and 21 excluded).

The participants received study medication in a standing position, followed by 20 min sitting upright, and then supine position until the end of the 8^th^ h; they were allowed to get out of bed for toileting needs.

Blood samples were taken, and supine BP and heart rate (HR) were measured by an automated device at the indicated time points for a period of 24 h after drug ingestion, during which they remained in the study unit. Subjects stayed in a supine position for the first 7 h and rested for at least 5 min in the supine position for all measurements thereafter. Data are reported as means ± SD for group means and as effect sizes with their 95% confidence intervals. Group differences were analyzed by two-tailed t-tests assuming equal variability in both groups; based on the exploratory character of a post hoc analysis, the calculated *p*-values should be considered as descriptive and not as hypothesis testing.

Venous blood samples (4 mL) were obtained at the indicated time points, placed on ice, and centrifuged within 1 h to obtain plasma, which was stored at -20° C until transfer to the bioanalytical lab. All analyses were performed according to the European Medicines Agency guideline for Bioanalytical Method Validation and standard operating procedures of Parexel BASD (Bloemfontein, South Africa).

According to the study protocol, participants were supposed to refrain from intake of CAF for 24 h prior to 24 h after dosing. However, 10 participants were identified to have baseline plasma levels of CAF >10% of the observed C_max_ in the IBU/CAF arm (subjects 3, 12, 13, 14, 15, 16, 20, 22, 28, and 33). These subjects were excluded from the primary analysis, but their data are shown in the Online Supplement for full transparency. Moreover, eight participants (subjects 10, 12, 14, 15, 19, 22, 26, and 36) had a t_1/2_ > 8.5 h for CAF, indicating that they were slow metabolizers. An exploratory analysis comparing CAF effects in normal *vs*. slow metabolizers among those without protocol violation is shown in the Online Supplement.

## 3 RESULTS

As previously reported [6], intake of IBU/CAF resulted in a CAF exposure (AUC_0-∞_ of 19.87 ± 7.79 µg·h/mL) and a C_max_ of 2.15 ± 0.44 µg/mL.

**Fig. (1):**
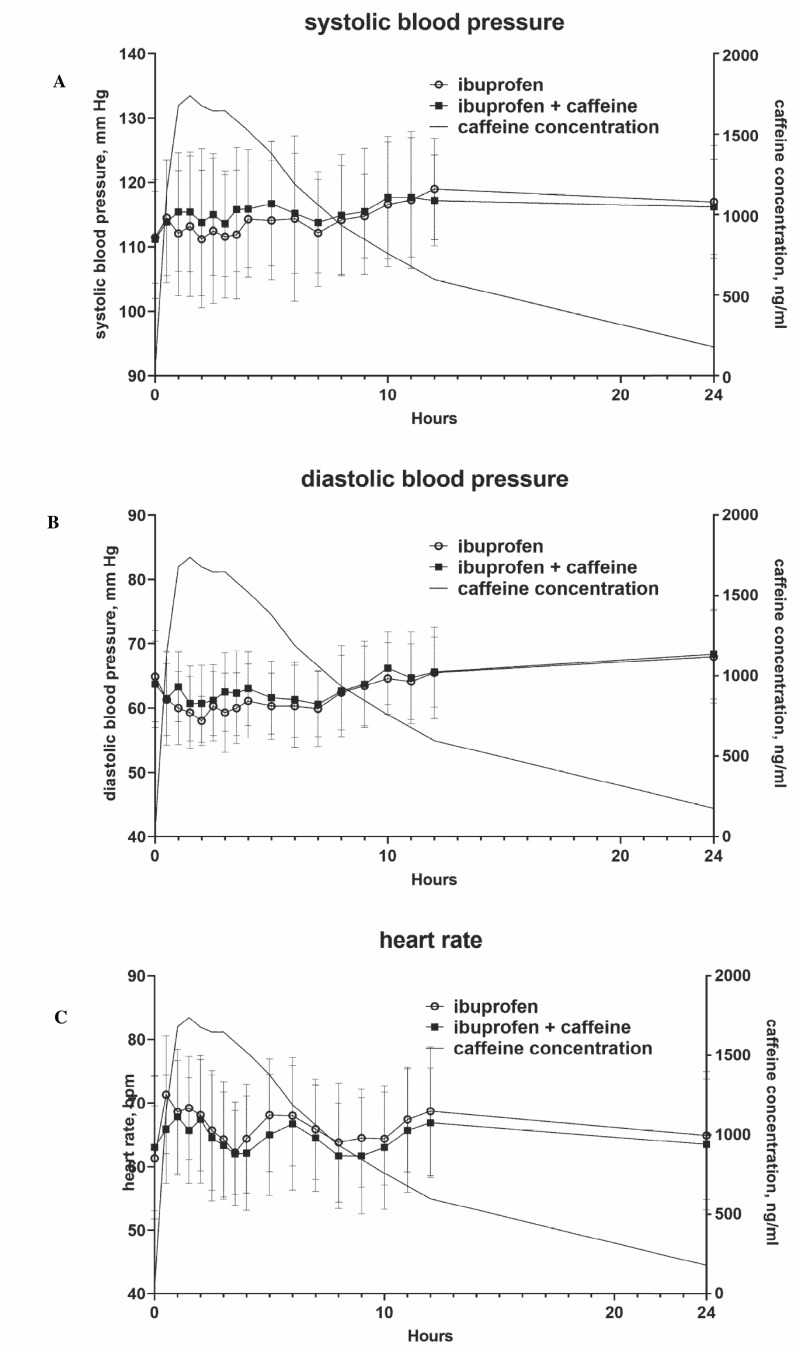
(**A-C**) Time course of vital signs over 24 h in the absence and presence of CAF ingestion. Data are means ± SD of 26 subjects. Data on corresponding plasma concentrations of CAF, as reported previously [6], are overlaid. An enlarged version focusing on the first 6 h after administration is shown in the Fig. (**S3**).

Mean systolic and diastolic BP (SBP and DBP, respectively) at baseline were 111.5 ±7.1 *vs*. 111.2 ± 9.2 mm Hg and 64.9 ± 7.1 *vs*. 63.7 ± 6.7 mm Hg in the absence and presence of CAF. Mean HR was 61.3 ± 8.3 *vs*. 63.0 ± 11.3 bpm. SBP, DBP, and HR curves over time were almost superimposable in the two groups, including time points of peak plasma concentrations of CAF (Fig. **1**). To increase the robustness of the analysis, we calculated intraindividual changes in SBP from baseline and averaged those over the 24-h observation period for each subject; these were 2.6 ± 6.0 and 4.3 ± 6.1 mm Hg in the absence and presence of CAF (mean difference 1.8 mm Hg [95% confidence interval -1.6; 5.^2^], *p* = 0.2918). CAF metabolizer status had no apparent effect on vital signs in either the control or the CAF period (Figs. **S1** and **S2**).

**Fig. (2):**
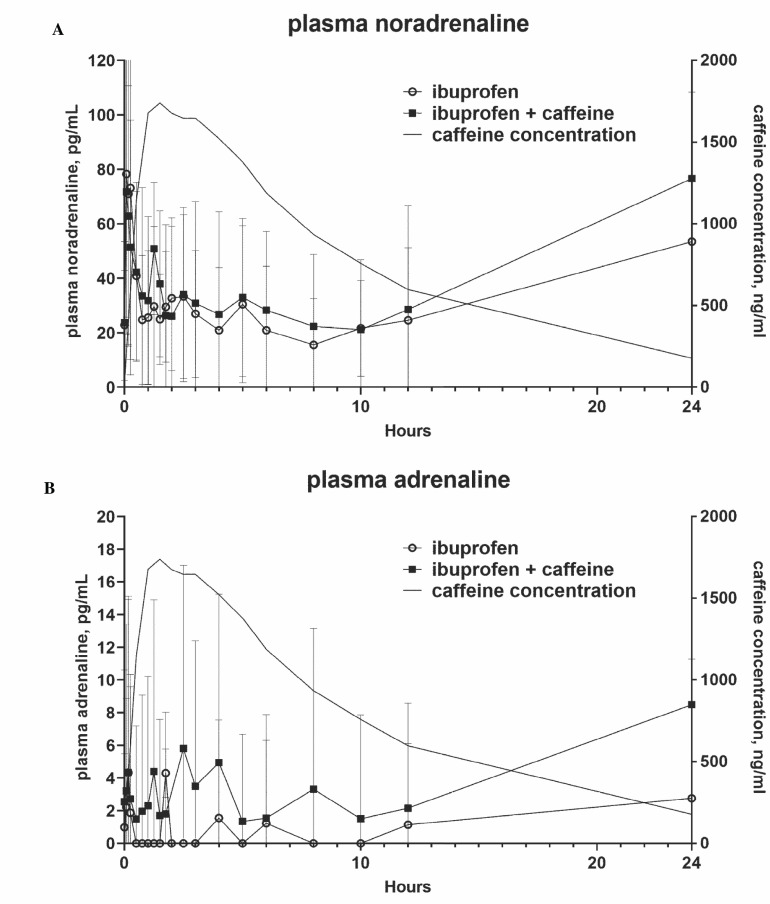
(**A** and **B**) Time course of plasma levels of noradrenaline and adrenaline over 24 h in the absence and presence of CAF ingestion. Data are means ± SD of 12-21 subjects (no catecholamine data available for some subjects at some time points in both treatment periods). Data on corresponding plasma concentrations of CAF, as reported previously [6], are overlaid.

The plasma levels of noradrenaline increased from a baseline of about 20 pg/mL to about 80 pg/mL 5 min after drug ingestion and then were stable at levels of about 20-30 pg/mL for the next 12 h (Fig. **2**). Plasma levels of adrenaline were very low (mostly below 10 pg/mL) and median values were 0 (below detection limits) at most time points. More importantly, the time courses of both catecholamines during the 24-h observation period in the absence and presence of CAF were superimposable (Fig. **2**) and the initial surges preceded that of CAF appearance in the general circulation, indicating that they were related to posture change and not a reaction to the administration of CAF.

## 4 DISCUSSION

### 4.1 Critique of Methods

The present manuscript presents a post hoc analysis of a previously reported bioequivalence study. While vital signs and plasma catecholamines had been collected according to a prespecified protocol, the study had not primarily been designed for their assessment. As the IBU/CAF fixed-dose combination is approved for occasional use, we had chosen to analyze data from a single-dose study.

The IBU/CAF fixed-dose combination contains 100 mg CAF. A comparison with the CAF intake in caffeinated beverages is difficult because, *e.g*., caffeinated beverages have been reported to contain 30-1780 mg/L CAF [7]. However, based on most analyses, 100 mg CAF can be considered at the low end of what is reported to be contained in a serving of a caffeinated beverage. The observed CAF plasma concentrations in the present study were in line with those reported by others [8], thereby validating our analytical approach.

Despite numerous studies, the interpretation of existing data on a possible relationship between CAF exposure and BP remains difficult due to several factors [4]. Firstly, BP is regulated by the interplay of many variables, including age, gender, genetics, and lifestyle factors. Therefore, we applied a cross-over design to minimize the effect of such variables. Specifically, differences in CAF metabolism, most likely due to polymorphisms in the CYP 1A2 gene, may lead to different exposures over time to the same CAF ingestion. Therefore, we have done an exploratory analysis to compare vital signs in both treatment periods between subjects with normal and slow CAF metabolism; this yielded no apparent difference (Online Supplementary Fig. **S2**). Similarly, CAF intake was unrelated to cardiovascular risk in a large previous study also when a polymorphism in the CYP 1A2 gene was taken into consideration [9]. Second, large studies have typically estimated CAF ingestion based on self-reported intake of type and quantity of caffeinated beverages, which provides imprecise information on true intake. Third, the acute effects of CAF intake are transient [10] and may not translate into those observed upon chronic/habitual use of caffeinated beverages, according to a meta-analysis [3].

When taken chronically in high doses (for instance, 600-800 mg thrice daily), IBU alone can increase BP [11, 12], whereas such effects are uncommon for lower doses taken for a short period. While the present study has not tested IBU alone *vs*. placebo, the lack of vital sign changes relative to baseline (other than expected diurnal profiles) is in line with these previous findings.

### 4.2 Caffeine Effects on Blood Pressure and Catecholamines

Minor and transient elevations of SBP (3-5 mm Hg, *i.e*., about 30-50% of SD population) have been reported in studies with acute ingestion of 160 mg CAF in healthy subjects aged 20-55 years [10]. The IBU/CAF fixed-dose combination contained a smaller dose of CAF (100 mg) and was associated with an even smaller numerical SBP difference (1.8 mm Hg), but the corresponding 95% confidence interval included 0. Similarly, small and transient SBP increases were seen in subjects not habitually drinking coffee but absent in habitual drinkers [13]. However, these data do not allow excluding that a different situation may exist in subjects with uncontrolled hypertension. On the other hand, in a cross-sectional study with 24 h ambulatory blood pressure monitoring in 715 elderly (≥63 years) hypertensive patients, those ingesting ≥3 cups/day had a 3.3 and 2.2 mm Hg greater SBP and DBP, respectively, compared to non-drinkers while smaller if any elevations were found in those ingesting 1 or 2 cups/day [14]. Taken together, previous studies under laboratory conditions and large clinical studies consistently show that a dose of 100 mg CAF causes little elevation of BP. The present data demonstrating a lack of effect of CAF on plasma catecholamine levels mechanistically support the idea that occasional intake of 100 mg CAF has little effect on vital signs.

Accordingly, meta-analysis has shown that habitual coffee drinking was not associated with an increased BP or cardiovascular risk [3]: five trials with acute administration of 200-300 mg CAF (2-3 time dose of an IBU/CAF tablet) reported an SBP elevation by 8.1 mm Hg, typically occurring within the first hour after intake; in contrast, no change in SBP was detected in three 2-week studies or in seven large cohort studies [3]. Accordingly, three randomized controlled trials using the present IBU/CAF fixed-dose combination with doses up to 1200/300 mg for up to 6 days and a total of 539 exposed patients reported only a single (mild) cardiovascular adverse event, a case of palpitation ([15, 16] and data on file).

A more recently reported, biobank-based study analyzed 347,077 subjects, including 8369 incident cardiovascular disease cases and compared habitual coffee drinkers (1-2 cups/day) to non-drinkers, drinkers of decaffeinated coffee, and heavy users (>6 cups/day) [9]: interestingly, non-drinkers, drinkers of decaffeinated coffee, and heavy users had an increase of cardiovascular risk by 11%, 7%, and 22%. Accordingly, the European Food Safety Authority has concluded, based on a comprehensive analysis of the available evidence, that CAF intake by adults of up to 3 mg/kg is of no concern [7].

Taken together, these data suggest that BP elevation and cardiovascular risk increases are limited to those with a chronic CAF intake being at least three times that of an IBU/CAF tablet. Moreover, studies with acute administration of CAF probably overestimate the cardiovascular effects of small to medium doses of CAF due to the transient nature of these effects. Therefore, our study design should have been very sensitive to detect BP alterations associated with acute CAF ingestion.

## CONCLUSION

A dose of 100 mg CAF as contained in a fixed-dose combination of IBU/CAF did not affect vital signs in healthy volunteers as observed over a 24-h period; this similarly applied to subjects with slow CAF metabolism. These data do not support the hypothesis that occasional intake of a small dose of CAF as part of pain medication imposes a health risk. Based on the proven increase in efficacy, the addition of CAF to non-opioid analgesics such as IBU has a favorable risk/benefit profile for occasional use. While we consider it likely that our observations are also applicable to repeated use, this requires further investigation.

## Data Availability

Qualified researchers may request access to patient-level data and related study documents, including the clinical study report, study protocol with any amendments, blank case report form, statistical analysis plan, and data set specifications. Patient-level data will be anonymized, and study documents will be redacted to protect the privacy of trial participants. Further details on Sanofi’s data-sharing criteria, eligible studies, and the process for requesting access can be found at https://www.clinicalstudydatarequest.com.

## SUPPLEMENTARY MATERIAL

Data comparing vital signs in per protocol subjects and protocol violators (pre-dosing intake of CAF) and between subjects with normal and slow CAF metabolism are shown in the Online Supplement. Supplementary material is available on the publisher’s website along with the published article.

## References

[1] Lipton R.B., Diener H-C., Robbins M.S., Garas S.Y., Patel K. (2017). Caffeine in the management of patients with headache.. J. Headache Pain.

[2] Derry CJ, Derry S, Moore RA (2014). Caffeine as an analgesic adjuvant for acute pain in adults. Cochrane Database of Systematic Reviews.

[3] Mesas A.E., Leon-Muñoz L.M., Rodriguez-Artalejo F., Lopez-Garcia E. (2011). The effect of coffee on blood pressure and cardiovascular disease in hypertensive individuals: A systematic review and meta-analysis.. Am. J. Clin. Nutr..

[4] De Giuseppe R., Di Napoli I., Granata F., Mottolese A., Cena H. (2019). Caffeine and blood pressure: A critical review perspective.. Nutr. Res. Rev..

[5] Derry S, Wiffen PJ, Moore RA (2015). Single dose oral ibuprofen plus caffeine for acute postoperative pain in adults. Cochrane Database of Systematic Reviews.

[6] Weiser T., Schepers C., Mück T., Lange R. (2019). Pharmacokinetic properties of ibuprofen (IBU) from the fixed-dose combination IBU/caffeine (400/100 mg; FDC) in comparison with 400 mg IBU as acid or lysinate under fasted and fed conditions—data from 2 single-center, single-dose, randomized crossover studies in healthy volunteers.. Clin. Pharmacol. Drug Dev..

[7] European Food Safety Authority (2015). Scientific opinion on the safety of caffeine.. EFSA J..

[8] White J.R., Padowski J.M., Zhong Y. (2016). Pharmacokinetic analysis and comparison of caffeine administered rapidly or slowly in coffee chilled or hot versus chilled energy drink in healthy young adults.. Clin. Toxicol. (Phila.).

[9] Zhou A., Hyppönen E. (2019). Long-term coffee consumption, caffeine metabolism genetics, and risk of cardiovascular disease: A prospective analysis of up to 347,077 individuals and 8368 cases.. Am. J. Clin. Nutr..

[10] Papakonstantinou E, Kechribari I (2016). Sotirakoglou Κ, et al. Acute effects of coffee consumption on self-reported gastrointestinal symptoms, blood pressure and stress indices in healthy individuals.. Nutr. J..

[11] MacDonald T.M., Richard D., Lheritier K., Krammer G. (2010). The effects of lumiracoxib 100 mg once daily vs. ibuprofen 600 mg three times daily on the blood pressure profiles of hypertensive osteoarthritis patients taking different classes of antihypertensive agents.. Int. J. Clin. Pract..

[12] Ruschitzka F., Borer J.S., Krum H. (2017). Differential blood pressure effects of ibuprofen, naproxen, and celecoxib in patients with arthritis: The PRECISION-ABPM (Prospective Randomized Evaluation of Celecoxib Integrated Safety Versus Ibuprofen or Naproxen Ambulatory Blood Pressure Measurement) Trial.. Eur. Heart J..

[13] Zimmermann-Viehoff F., Thayer J., Koenig J., Herrmann C., Weber C.S., Deter H.C. (2016). Short-term effects of espresso coffee on heart rate variability and blood pressure in habitual and non-habitual coffee consumers--a randomized crossover study.. Nutr. Neurosci..

[14] Lopez-Garcia E., Orozco-Arbeláez E., Leon-Muñoz L.M. (2016). Habitual coffee consumption and 24-h blood pressure control in older adults with hypertension.. Clin. Nutr..

[15] Weiser T., Richter E., Hegewisch A., Muse D.D., Lange R. (2018). Efficacy and safety of a fixed-dose combination of ibuprofen and caffeine in the management of moderate to severe dental pain after third molar extraction.. Eur. J. Pain.

[16] Predel H.G., Ebel-Bitoun C., Lange R., Weiser T. (2019). A randomized, placebo- and active-controlled, multi-country, multi-center parallel group trial to evaluate the efficacy and safety of a fixed-dose combination of 400 mg ibuprofen and 100 mg caffeine compared with ibuprofen 400 mg and placebo in patients with acute lower back or neck pain.. J. Pain Res..

