# Supplementary material for: Lack of Evidence for Blood Pressure Effects of Caffeine Added to Ibuprofen
Source: Curr Drug Saf. 2023 Jan 1;18(1):97–102. doi: 10.2174/1574886317666220414125027 (PMC10173466; doi:10.2174/1574886317666220414125027)
Supplement: Supplementary file 1 [file CDS-18-97_SD1.pdf]

Supplementary Material

Lack of Evidence for Blood Pressure Effects of Caffeine Added to Ibuprofen

Anette Lampert[1], Robert Lange[1] and Thomas Weiser[1,*]

[1]*CHC Medical Affairs, Sanofi-Aventis Deutschland GmbH, Frankfurt am Main, Germany*

This Online Supplement describes additional exploratory analyses comparing subjects who had complied with the study protocol to abstain from intake of caffeine for 24 h prior to dosing (per protocol) with those exhibiting pre-dose caffeine concentrations of >10% of peak values (protocol violators). It also describes a comparison for per protocol subjects considered to be normal as compared to slow caffeine metabolizers ($t_{1/2}$ > 8.5 h).

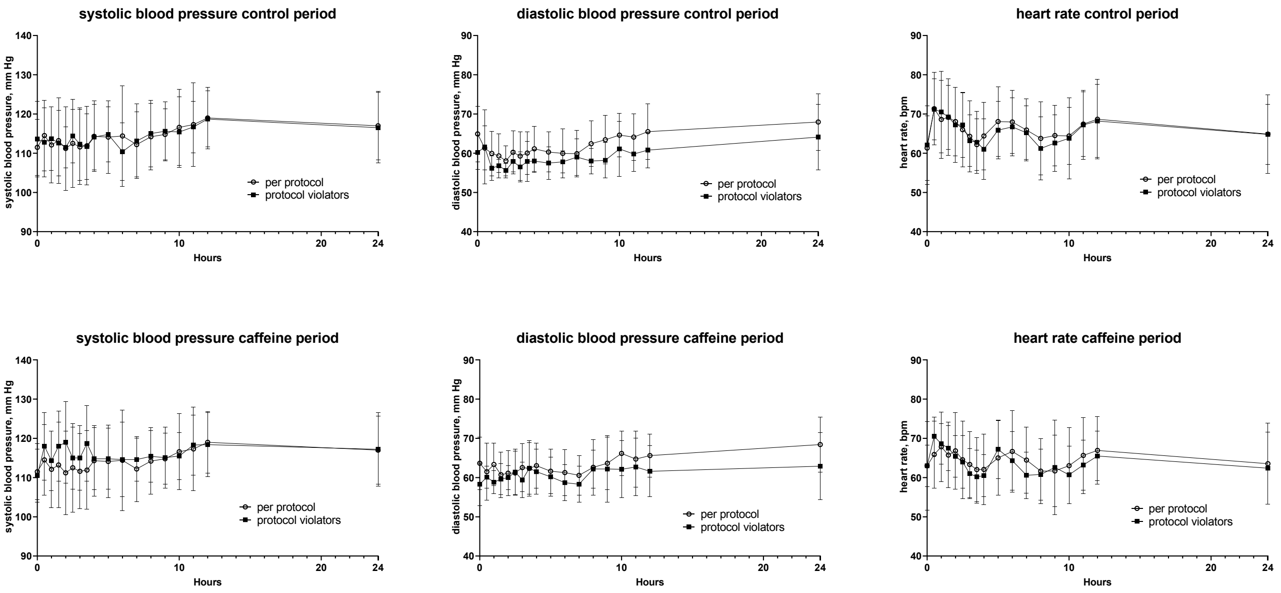

Supplementary Figure 1: Systolic and diastolic blood pressure and heart rate (left, middle and right columns, respectively) during the control and caffeine period (upper and lower row, respectively) in per protocol subjects (n = 26, open symbols) and the protocol violators (detectable caffeine levels prior to dosing; n = 10, filled symbols).

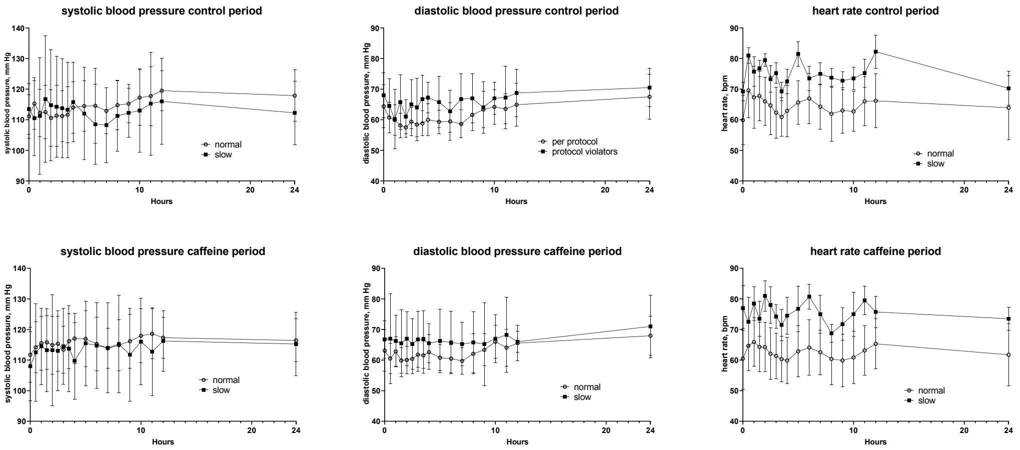

Supplementary Figure 2: Systolic and diastolic blood pressure and heart rate (left, middle and right columns, respectively) during the control and caffeine period (upper and lower row, respectively) in per protocol subjects with normal caffeine metabolism (n = 22, open symbols) and with slow caffeine metabolism ($t_{1/2} > 8.5$ h; n = 4, filled symbols). Note that an apparently higher heart rate in slow metabolizers in both treatment periods was also present at the time point immediately before drug intake, i.e., represented a feature of this small subset of subjects, not an enlarged response to caffeine.

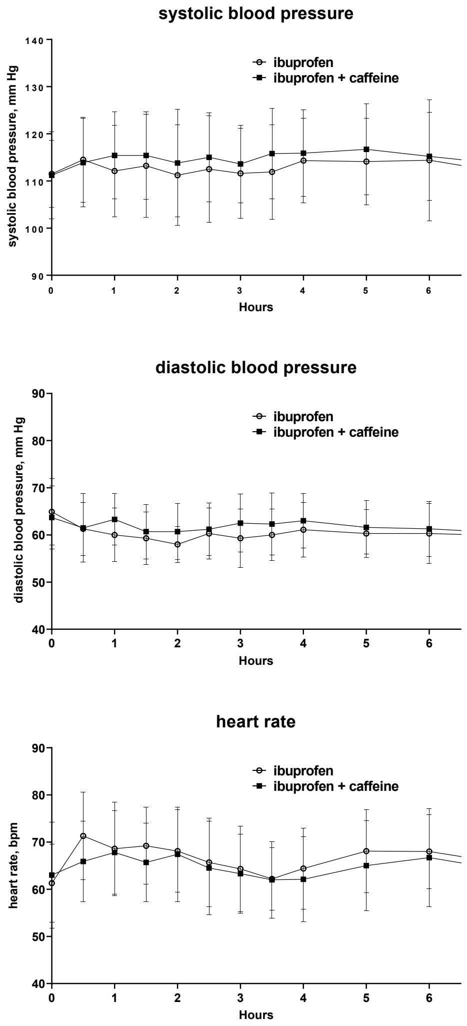

Supplementary Figure 3: Time course of vital signs in the absence and presence of CAF ingestion. Data are means ± SD of 26 subjects. Data are identical to those shown in Figure 1 of the main paper except that now only the first 6 h are shown to enable better inspection for this time frame.
